# OLIGOMETASTASIS IN GASTRIC CANCER TREATMENT: IS THERE A PLACE FOR THE SURGEON?

**DOI:** 10.1590/0102-672020230034e1752

**Published:** 2023-09-15

**Authors:** Paulo Pimentel de ASSUMPÇÃO, Jéssica Manoelli Costa da SILVA, Danielle Queiroz CALCAGNO, Williams Fernandes BARRA, Geraldo ISHAK, Paulo KASSAB

**Affiliations:** 1 Universidade Federal do Pará, Oncology Research Center – Belém (PA), Brazil; 2 Universidade Federal do Pará, João de Barros Barreto University Hospital, General Surgery and Digestive Tract Service – Belém (PA), Brazil; 3 Faculdade de Ciências Médicas da Santa Casa de São Paulo, Department of Surgery – São Paulo (SP), Brazil

**Keywords:** Stomach Neoplasms, Neoplasm Metastasis, General Surgery, Chemoradiotherapy, Neoplasias Gástricas, Metástase Neoplásica, Cirurgia Geral, Quimiorradioterapia

## Abstract

Metastatic gastric cancer traditionally hinders surgical treatment options, confining them to palliative procedures. The presence of metastases in these tumors is classified as M1, irrespective of their characteristics, quantity, or location. However, oligometastatic disease emerged as an intermediate state between localized and widely disseminated cancer. It exhibits diverse patterns based on metastatic disease extent, type, and location. Adequately addressing this distinctive metastatic state necessitates tailored strategies that surpass the realm of palliative care. Differentprimary tumor types present discernible scenarios of oligometastatic disease, including preferred sites of occurrence and chronological progression. Due to the novelty of this theme and the heterogeneity of the disease, uncertainties still exist, and the ability to provide confident guidelines is challenging. Currently, there are no effective predictors to determine the response and provide clear indications for surgical interventions and systemic treatments in oligometastatic disease. Treatment decisions are commonly based on apparent disease control by systemic therapies, with a short observation period and imaging assessments. Nonetheless, the inherent risk of misinterpretation remains a constant concern. The emergence of novel technologies and therapeutic modalities, such as immunotherapy, cellular therapy, and adoptive therapies, holds the potential to reshape the landscape of surgical treatment for the oligometastatic disease in gastric cancer, expanding the surgeon’s role in this multidisciplinary approach. Prospective tools for patient selection in oligometastatic gastric cancer are being explored. Using non-invasive, cost-effective, widely available imaging techniques that provide real-time information may revolutionize medical practice, ensuring precision medicine accessibility, even in resource-constrained small healthcare facilities. Incorporating molecular classifications, liquid biopsies, and radiomic analysis in a complementary protocol will augment patient selection precision for surgical intervention in oligometastasis. Hopefully, these advancements will render surgeries unnecessary in many cases by providing highly effective alternative treatments.

## RESUMO

O câncer gástrico metastático representa um desafio para o tratamento cirúrgico, restringindo-se a procedimentos paliativos. A presença de metástases nestes tumores é categorizada como estágio M1, independentemente das características, quantidade e localização. No entanto, a doença oligometastática surgiu como um estado intermediário entre o câncer localizado e o amplamente disseminado. A oligometastática apresenta diversos padrões com base na extensão, tipo e localização da doença metastática. Abordar adequadamente esse estado distintivo requer estratégias adaptadas que ultrapassem o escopo dos cuidados paliativos. Diferentes tipos de tumores primários exibem cenários distintos de oligometastática, incluindo locais preferenciais de ocorrência e progressão cronológica. Devido à novidade desse tema e à heterogeneidade da doença, ainda existem incertezas, e a capacidade de fornecer diretrizes seguras é limitada. Atualmente, não existem preditores eficazes para determinar a resposta e fornecer indicações claras para intervenções cirúrgicas e tratamentos sistêmicos em oligometastática. As decisões de tratamento geralmente se baseiam no controle aparente da doença por meio de terapias sistêmicas, com um curto período de observação e avaliação por imagem. No entanto, o risco inerente de interpretação incorreta continua sendo uma preocupação constante. A emergência de novas tecnologias e modalidades terapêuticas, como imunoterapia, terapia celular e terapias adotivas, tem o potencial de remodelar o panorama do tratamento cirúrgico da oligometastática no câncer gástrico, expandindo o papel do cirurgião nessa abordagem multidisciplinar. Ferramentas prospectivas para a seleção de pacientes com câncer gástrico oligometastático estão sendo exploradas. A utilização de técnicas de imagem não invasivas, rentáveis e amplamente disponíveis, que fornecem informações em tempo real, pode revolucionar a prática médica, garantindo a acessibilidade da medicina de precisão, mesmo em unidades de saúde com recursos limitados. A incorporação de classificações moleculares, biópsias líquidas e análises radiômicas em um protocolo complementar aumentará a precisão da seleção de pacientes para intervenção cirúrgica em oligometástases. Espera-se que esses avanços tornem as cirurgias desnecessárias em muitos casos, proporcionando tratamentos alternativos altamente eficazes.

## INTRODUCTION

Metastases in gastric cancer used to be a barrier for surgical treatment and restricted to palliative procedures^23^. The presence of metastases in these tumors is considered M1, regardless of their type, number, or site. Different M1 used to be taken as a unique clinical condition. Conversely, there are plenty of different scenarios considering the amount, type, and site of metastatic diseases^4,11^.

The concept of oligometastatic disease (OMD) was first reported in an editorial of the Journal of Clinical Oncology as an intermediate state between locally restricted and widely disseminated cancer^20^. This clinical condition should be considered a peculiar metastasis state requiring specific approaches that might include treatments beyond palliation^28,44^.

OMD has different patterns according to the primary tumor type, including preferable specific sites of occurrence and chronological order. Recently, a consensus was published to establish clear concepts and guidelines on investigations and management of these clinical conditions. Besides, oligometastatic gastric cancer (OMGC) also demanded specific consensus and definitions not covered by this general document^19^.

In 2022, the OligoMetastatic Esophagogastric Cancer (OMEC) project was launched in Europe as an ambitious initiative to be developed by a collaborative group involving most of the related scientific societies and many countries in that continent^29^. Initially, the OMEC project published a systematic literature revision with meta-analysis, providing preliminary concepts and checking the potential impacts of offering local treatment for OMD^28^.

**Figure 1:**
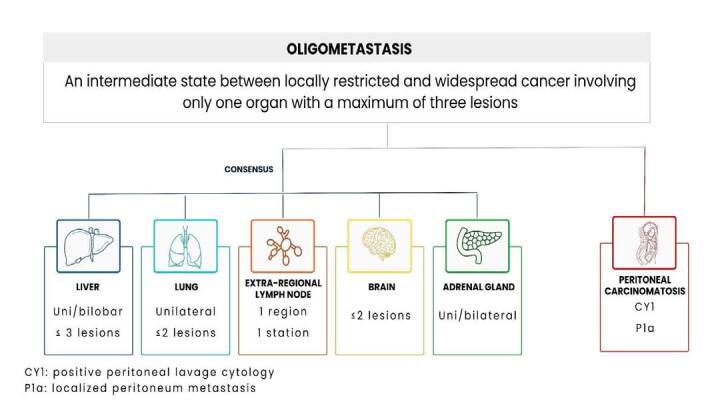
Definition of oligometastatic disease and the most common sites of metastases in gastric cancer.

Among the proposed definitions, the diagnosis of OMD should be performed by computed tomography (CT) scan, magnetic resonance imaging (MRI), or positron emission tomography CT (PET-CT) images, and the maximum number of metastases should be limited to three. Only one site was admitted for CT of distant metastases, and restrictions were also specified peculiarly for each metastatic site (Figure 1)^28^.

According to this meta-analysis, the association between metastasis local treatment and systemic chemotherapy yielded the most effective outcomes, though shedding light on the potential benefits of surgical interventions beyond palliation. Nevertheless, since these novel concepts have been constructed by literature review only, the possibility of misinterpretations and biases was reported, and additional studies were recommended^28^.

Moreover, another study by the same team examined the results of different approaches to treating OMGC. As a result, local plus systemic therapy was considered the best support or the greatest treatment option compared to local alone or systemic alone. Once again, this conclusion must be taken with prudence since it was a retrospective study with no control of the groups, and thus at risk of bias^25^.

Finally, to find out what would happen in a real-life scenario, clinical cases of metastatic gastric cancer were discussed in tumor board events among different centers and teams. Overall,there was agreement on the concept of oligometastasis (the same as that from the previous consensus paper), on the re-stage of patients after neoadjuvant chemotherapy (CT, MRI, or PET-CT scans), and regarding the definition of OMD after treatment (with no new lesions, even admitting an increase in pre-existing metastases)^27^.

Despite consensus on an initial approach to characterize, stage, and re-check clinical responses after new adjuvant chemotherapy, the final decision towards operating the OMGC was not consensual, exposing the persistence of controversies and the fragility of the proposed recommendations^27^.

The novelty of the theme and the disease heterogeneity contributes to these uncertainties. There are no effective predictors of response able to allow clear indications for surgical interventions and system treatments.Most importantly, no available randomized clinical trial results define clear indications and benefits for treating oligometastasis in GC^11,18^.

The REGATTA trial, which originated in Asia and was published in 2016, provoked a misinterpretation of the surgery’s role in treating OMGC.This phase III randomized trial compared chemotherapy alone versus surgery followed by chemotherapy in cases of unique metastasis of GC. According to the interim analyses, the trial was interrupted due to not reaching the expected benefits in the surgical group. The conclusions suggested the refuting of surgical treatment in the presence of oligometastasis, which was reported and followed worldwide for many years^17^.

Nevertheless, criticism still exists regarding that trial design. The metastases were not resected in the surgical arm protocol, and the surgical procedure regarding the primary treatment was limited to a D1 lymphadenectomy. In simple words, the metastases were not treated, and the primary tumors were inadequately treated, since a D2 lymphadenectomy is the standard procedure according to what is proposed by the current guidelines. Furthermore, unlike the current recommendations, chemotherapy was indicated post-operatively instead of in a neoadjuvant approach. Hence,in conditions that differ from current surgical protocols, the indication for surgical treatment of oligometastasis would not be supported^17,22,32^.

The consequences of misinterpreting the REGATTA trial results were extremely serious. Any surgical procedure beyond palliation has been deemed an aberration for many years. However, careful interpretation of REGATTA trial data paved the way for further investigations, correcting deviations that could impact the outcomes and conclusions.

Among these new interventions, the FLOT-3 trial, a German phase II randomized trial, compared the neoadjuvant (instead of adjuvant chemotherapy as done in the REGATTA trial) FLOT chemotherapy plus surgery versus exclusive chemotherapy in treating oligometastatic gastric or esophagogastric cancers^3^. The results favored the surgical group, presenting better overall and disease-free survival rates, differently from the findings of the REGATTA trial. Due to these relevant and confronting results, the FLOT-3 trial results supported the proposal of another phase III trial, the FLOT-5^11^.

The FLOT-5 trial was designed similarly to the FLOT-3. Surgery was preceded by neoadjuvant FLOT chemotherapy. This trial also included evaluating the quality of life as an additional endpoint.Only oligometastatic cases were selected, naïve of clinical and surgical treatment, in patients with good performance status. Both primary and metastatic tumors must be potentially resectable. After receiving four cycles of FLOT chemotherapy, the patients were re-staged and, in case of not showing disease progression, they were randomized to surgery, including metastasis and primary tumor treatment, or exclusive chemotherapy^2^. 

The results of FLOT-5 are awaited and expected to support new interpretations about the role of the surgery in OMGC. Other trials are also on track, including the evaluation of chemotherapy in an adjuvant scenario^11,24^.

In addition, peritoneal carcinomatosis poses a critical challenge. Although not included in most accepted criteria for oligometastasis, the frequent presence of multiple implants and limited diagnostic capabilities through image-guided methods make it a common type of metastasis that needs to be addressed^5,18,42^.

Some clinical trials include minimal peritoneal disease represented by cytology-only positivity or few peri-gastric implants, together with classical oligometastasis. This is performed to determine whether the limited peritoneal diseases can be managed as oligometastasis^2,18^.

Preliminary results from a small series of non-controlled studies favor the inclusion of such peculiar situations as possible clinical indications for systemic plus surgical treatment, depending on the confirmation of the results from randomized trials. Inthe absence of available results from the current well-designed clinical trials addressing the role of surgical treatment in GC oligometastasis, most critical scientific societies leading with GC treatment report weak recommendations on these themes^45^.

The International Gastric Cancer Association (IGCA) guidelines and The Brazilian Gastric Cancer Association (ABCG) guidelines, which were recently revised, have very stringent recommendations on the surgical approach to OMGC^6,41^.

Accordingly, in the IGCA guidelines, there are only three clinical conditions with low recommendations for surgicaltreatment:

1. few lymph nodes metastasis -16b (indicated only after new adjuvant treatment and with gastrectomy plus D2 lymphadenectomy plus para-aortic dissection);
2. only one liver metastasis; and
3. positive peritoneal cytology or limited peri-gastric implants.

Each of these conditions should be considered exclusively in case of being the unique site of metastasis. These recommendations are quite similar to that proposed by the ABCG guidelines^6,22^.

In 2021, Yoshida etal. published the Conversion Therapy for Stage IV Gastric Cancer 1 (CONVO-GC-1), an international retrospective cohort study, which indicated that the so-called “conversion therapy” could be an option in the metastatic scenario^48^. The sixth edition of the Japanese Gastric Cancer Treatment Guidelines also recommended conversion surgery after initial chemotherapy, although the level of evidence supporting this recommendation is relatively weak^22^.

### Conditional requirements for surgical treatment of oligometastasis in gastric cancer

The ongoing development of new technologies and approaches, such as immunotherapy, cellular therapy, and adoptive therapies, among others, may change the recommendations for surgical treatments of OMGC. Nevertheless,some necessary conditions must be followed when considering radical surgicalinterventions^11^.

Due to the complexity of treating GC with distant metastasis, the patient’s performance status needs to be checked, and only those able to withstand the proposed surgery will be selected.Surgeon experience is another requirement, as is the dynamic health support team and hospital excellence. Neoadjuvant treatment is essential. The response to pre-operative treatment determines whether to proceed to the surgical approach^9^. Defining the type of neoadjuvant treatment is a crucial step of the treatment plan and needs to address the gastric system disease^31^.

Surgery will be avoided in case of additional metastasis in the pre-operative setting, and another medical treatment might be considered. Precise reevaluation of the disease after neoadjuvant treatment is essential to avoid unnecessary surgical interventions. Currently, the response evaluation criteria in solid tumors (RECIST 1.1) are the preferred approach for reevaluation^16^. When staging the patient, the surgeon must have confidence that complete resection of both the primary tumor and the metastasis can be achieved. Reaching an R0 resection impacts the outcomes and must be addressed as a conditional indication^11^.

Several situations involving the treatment of OMGC entails more generalized recommendations. It is highly recommended that tumor board teams assess and discuss each case individually, considering the patient’s preferences. Nevertheless, choosing the optimal approach for synchronic liver metastasis, which require major hepatic resection, remains challenging in these complex scenarios^26^.

For non-symptomatic primary tumors, a liver-first approach might be considered, following an extended interval under systemic therapy, showing excellent disease control. Aftertreating the liver tumor, additional systemic therapy should be advised since the molecular microenvironment after a significant liver resection could favor dormant metastasis to progress^11,26,27^.

Primary tumor resection before addressing liver metastasis is an option. Nevertheless, in case of surgical complications, systemic therapy usually suffers large intervals of interruption that might impact the disease control.Patients with excellent clinical status may be considered for a concomitant approach to be discussed by highly experienced surgical teams. Alternatively,exclusive systemic therapy should be an option for some patients^11,27^.

Peritoneal carcinomatosis is a prevalent condition in GC but requires more precise recommendations. Althoughextrapolating the classical criteria for oligometastasis, the limited peritoneal disease is usually addressed together with classical OMGC^18^.

Considering non-macroscopic peritoneal disease, the cytology remains a fragile indication for clinical decisions among Western countries. Pathologists have differing views on the interpretation of peritoneal lavage samples due to many issues involving the quality of the sample preparation and the lack of expertise in these tricky analyses^5,47^.

The cytology reported during the surgical inventory is usually confirmed post-operatively, but this confirmation is expected to support a definitive decision. Most Western teams do not rely on cytology-only interpretation to choose definitive treatment strategies. On the other hand, positive cytology should indicate a systemic treatment and reevaluation after considering any surgical approach^45^.

If cases are converted to negative peritoneal disease after systemic therapy, radical treatment of the primary tumor may be considered. That is also the condition of minimal peri-gastric implants that reach complete response after neoadjuvant treatment; the disease that does not progress after systemic therapy is an eventual indication for concomitant treatment of the primary and peritoneal lesions in highly selected cases. Conversely, if peritoneal implants, including those restricted to the peri-gastric areas, appear after neoadjuvant treatment, another systemic strategy should be considered^37,45^.

Although strategies such as hyperthermic intraperitoneal chemotherapy (HIPEC) and pressurized intraperitoneal aerosol chemotherapy (PIPAC) have been explored, their benefits are still limited and associated with high morbidity. Thus, these approaches should be reserved for investigational protocols^12,14,46^.

**Figure 2:**
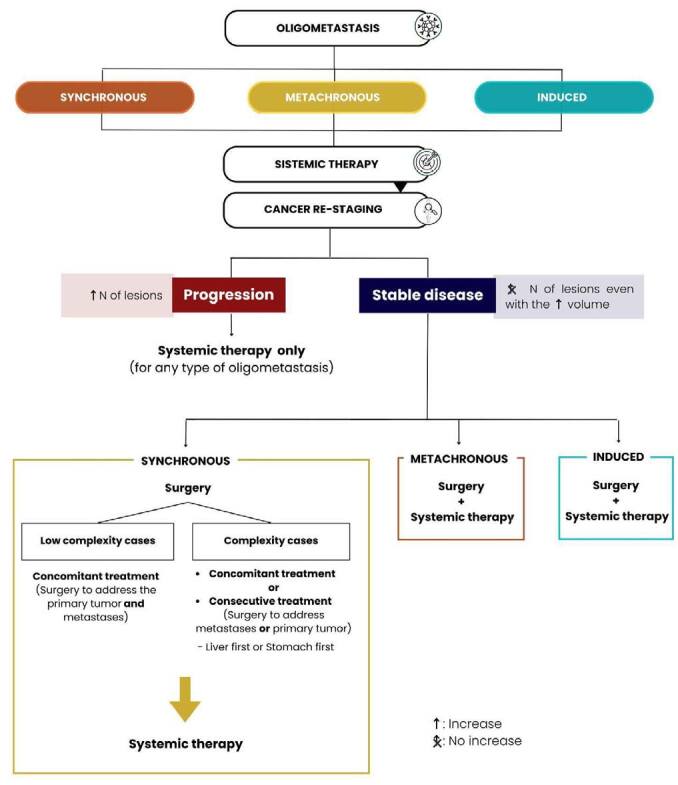
Essential steps in the management of oligometastatic gastric cancer.

Patients submitted to surgical treatment of OMGC that present oligometastatic recurrence, even after a long interval, will usually experience the disease progression, so the surgical approach should be avoided in most cases. Figure 2 summarizes the main steps and approaches to managing OMGC.

### Perspectives for better selecting patients for surgery

The decision to indicate a major and high-risk surgical intervention for patients with poor prognoses such as gastric adenocarcinoma, which additionally presents systemic metastasis, is extremely difficult to face^40^. The consequences of such a decision are potentially disastrous. If the decision is not to operate, the patient’s fate is a palliative treatment that might sound like a death sentence, with a short waiting time.On the contrary, a surgical indication may carry a considerable risk of complications and death. Besides, disease progression may occur even after a supposed curative surgery^31^.

The current understanding of OMGC is still fragile, and the diversity of this condition challenges the capacity to offer confident guidelines to be followed. The state of the art for treatment decision is based on an apparent control of the disease by systemic therapies, after a short interval of observation time, according to image stage status plus peritoneal checking. Nevertheless, misinterpretation is a real and constant possibility^11,24^.

Response to systemic therapy is tricky and may be transient. Occult metastasis may become apparent after the re-staging period. Diagnostic accuracy is also limited, even using modern CT, MRI, and PET-CT scans.Molecular tumor diversity is another important player that needs to be addressed^7^.

Although facing a dangerous enemy, innovations to deal with GC are on track. New systemic approaches, diagnostic tools, better molecular comprehension, and increments in surgical technique, including minimally invasive methods and devices, will favor better outcomes in the future. Some new potential selection tools for precise indications of surgical approaches in OMGC will be briefly discussed^13,31,33^.

### Selection according to molecular type

Among the fragilities in deciding the most effective treatments for OMGC, the absence of selection criteria based on molecular features stands out.The advances in understanding the molecular patterns of many cancers raised innovations in their management. The Cancer Genome Atlas (TCGA) molecular classification has proposed four types of GC, each presenting specific characteristics that might influence prognosis and response to therapies^8^.

Accordingly, Genomically Stable (GE) GC type presents the worst prognosis due to high aggressiveness and poor responses to systemic therapy. The absence of molecular targets for therapy and non-responsiveness to immune checkpoints blockage in this case make the scenario even worse. The molecular aspect of moving from an epithelial-like to a mesenchymal-like pattern – the epithelial-mesenchymal transition – favors metastatic dissemination. This condition associated with the scarcity of efficient systemic therapies, in theory, puts this GC type as the one with the weakest benefit for oligometastasis aggressive surgical treatment^8^.

The Microsatellite Instability (MSI) and the Epstein-Barr Virus (EBV) types keep epithelial patterns and are responsive to immunotherapies, even in the metastatic status. These types seem to be the most suitable for systemic plus surgical therapy in oligometastatic cases. Despite being a helpful indicator for managing oligometastasis, MSI types exhibit a limited response to fluoropyrimidines. Therefore, alternative systemic regimens, including immunotherapy, should be considered potential treatment options. In some thoroughly selected cases, upfront surgery may be feasible^1,8,39^. 

Chromosomal Instability (CIN) GC presents intermediary patterns compared to GE versus MSI and EBV types. There are potential targets for therapy, although present in a minority of cases, and selection for oligometastasis treatment should be performed individually^21^.

The TCGA molecular classification was not utilized to inform clinical decisions in the current reports on oligometastasis treatment for gastric cancer. However, retrospective analysis of these series can be performed to shed light on the relevance of the molecular types in determining the management of OMGC.

### The evaluation of oligometastasis images

The concepts of OMD and its re-staging after neoadjuvant chemotherapy are based on classical CT, MRI, or PET-CT images^27^. Nevertheless, the information from these methods was limited to the existence, dimensions, and number of metastases. Prognosis and treatment responsiveness is not accurately addressed, leaving crucial components for clinical decisions out.

Radiomics are coming to enhance the power of medical images by adding putative molecular pattern information to conventional image features, contributing to favorable clinical decisions^10,30^.

The immune status defined by radiomics reproduces that of immunohistochemistry assays. If confirmed in large series and by using widely available and reproducible methods, the capacity to indicate gene expression patterns will promote a revolution in pre-operative medical decisions^36^.

Obtaining tissue samples and performing molecular investigations takes time, effort, and financial support. Thepossibility of inferring molecular patterns using non-invasive, cost-effective, widely available images in almost real-time will change medical practice and provide precision medicine accessibility. Imageanalysis could be performed from external specialized centers, helping hospitals in small communities with low costs while making it more familiar to less experienced radiologists.

Attempts to confirm the real power of this new technology can also take advantage of available data from previous studies. Archival images can be confronted with molecular and clinical data and outcomes, favoring discoveries and improvements and reducing clinical translation time^10,15,43^.

### Liquid biopsy

The next step in oncology investigations on track to come to clinical practice is the liquid biopsy, a minimally invasive test to identify tumor components in human fluids. The most used one is blood test, that can search for different tumor components, including mainly circulating tumor cells (CTC) and cell-free circulating DNA fragments (ctDNA)^33,38^.

Some commercial blood tests are available, including panels for molecular abnormalities commonly found in specific tumor types, like colon cancer and others.Regarding GC, these panels are tricky to construct due to the absence of typical molecular alterations resulting from the tumor heterogeneity. Even the most common molecular alterations cover less than 10% of cases requiring the construction of extensive and expensive panels.

Alternatively, sequencing the primary tumor, followed by choosing a few confirmed molecular signatures to be precisely targeted in the blood test of each patient, is an excellent choice to be applied individually, although at high cost. Some underdeveloped blood/liquid biopsies tests try to overcome the difficulties imposed by tumor heterogeneity. Among the most promising ones, are the analyses of patterns of DNA fragmentation that, if confirmed, may be applied to every type of tumor, favoring wide clinical application^34,35^.

Nevertheless, the ctDNA accuracy in GC carcinomatosis remains poorly investigated. Considering the celomic route of carcinomatosis, hypothetically, this type of metastasis does not need blood circulation to be implanted, and even products from tumor apoptosis occurring inside the peritoneal cavity, and coming to blood circulation, are yet to be confirmed as reliable markers for liquid biopsy. Alternatively, performing a liquid biopsy directly in peritoneal fluid or lavage might be an appropriate option^49^.

## CONCLUSIONS

OMGC is a potential indication for radical surgical treatment in selected cases. However, the process of case selection remains a challenge; the criteria are based on the response to neoadjuvant systemic treatments followed by a re-staging using conventional scans. Applying molecular classifications, liquid biopsies, and radiomic in a complementary protocol can greatly enhance the patient selection precision for oligometastasis surgical treatment. The integration of these innovative approaches and emerging treatment strategies holds promise for improving patient outcomes, refining indications for surgical interventions in OMGC, and ultimately reducing the necessity for surgical interventions in a significant proportion of cases.
